# Critical Insights Into the Management of Postpartum Left Main Spontaneous Coronary Artery Dissection: Current Strategies and Future Directions

**DOI:** 10.7759/cureus.44622

**Published:** 2023-09-03

**Authors:** Arya C Rathi, Nikhilesh Nagtode, Vaibhav Chandra, Aniket G Pathade, Seema Yelne

**Affiliations:** 1 Community Medicine, Jawaharlal Nehru Medical College, Datta Meghe Institute of Higher Education and Research, Wardha, IND; 2 Research and Development, Jawaharlal Nehru Medical College, Datta Meghe Institute of Higher Education and Research, Wardha, IND; 3 Nursing, Shalinitai Meghe College of Nursing, Datta Meghe Institute of Higher Education and Research, Wardha, IND

**Keywords:** collaborative research, multidisciplinary approach, diagnosis challenges, maternal health, management strategies, left main artery, spontaneous coronary artery dissection, postpartum

## Abstract

This review article delves into the multifaceted realm of postpartum left main spontaneous coronary artery dissection (PLMSCAD), an infrequent yet critical condition affecting women during the postpartum period. Through a comprehensive exploration of its pathophysiology, clinical presentation, diagnosis, management strategies, and future directions, this review provides a holistic understanding of PLMSCAD's complexities. The article highlights challenges in diagnosis due to overlapping symptoms and underscores the significance of prompt recognition and tailored interventions. Current management strategies, encompassing medical and interventional approaches, are analysed in the context of their short-term and long-term impact on patient outcomes. Ethical considerations and the role of patient education and support networks are explored, shedding light on the broader psychosocial dimensions of PLMSCAD management. As emerging research reveals insights into genetic influences, hormonal dynamics, and the prognosis of affected individuals, this review emphasises the necessity of collaborative research endeavours and data sharing to enhance our understanding and guide future strategies. Ultimately, this review underscores the urgency of addressing the unique needs of women experiencing PLMSCAD, urging ongoing research, multidisciplinary collaboration, and a patient-centred approach to optimise maternal health outcomes and well-being.

## Introduction and background

Postpartum left main spontaneous coronary artery dissection (PLMSCAD) is a rare and potentially life-threatening condition that occurs in women during the postpartum period. It is characterised by the spontaneous dissection of the coronary artery, particularly affecting the left main coronary artery. Spontaneous coronary artery dissection (SCAD) itself is an uncommon phenomenon. Still, when it occurs in the left main artery in the postpartum period, it presents unique challenges due to its sudden onset and potential for severe complications [1,2].

Maternal mortality and morbidity are critical concerns in obstetrics and gynaecology. Maternal mortality is unacceptably high. About 287,000 women died during and following pregnancy and childbirth in 2020 [3]. While advances in medical care have reduced overall maternal mortality, specific conditions such as PLMSCAD continue to pose a significant threat to maternal health. The postpartum period, often called the "fourth trimester," is a time of increased vulnerability for women due to the physiological changes and stresses associated with childbirth [3,4].

PLMSCAD can lead to acute myocardial infarction, heart failure, and sudden cardiac death. Recognising the importance of this topic is essential for healthcare providers to ensure timely and accurate diagnosis, appropriate management, and improved outcomes for affected women. By addressing the unique challenges PLMSCAD presents, clinicians can reduce maternal mortality rates and enhance postpartum care [5,6].

The primary objective of this review article is to consolidate and critically evaluate the existing knowledge on PLMSCAD, focusing on its clinical presentation, diagnosis, and management. This article comprehensively explains the current strategies for managing PLMSCAD cases by synthesising the available evidence. Furthermore, the review will explore potential gaps in the current approaches and propose directions for future research and clinical practices. In the face of limited data and the rarity of PLMSCAD cases, this article seeks to provide a valuable resource for healthcare professionals who encounter such cases in their clinical practice. By sharing insights into successful and challenging management strategies, the article aims to contribute to a broader dialogue on refining the care and outcomes of women affected by this complex and often life-altering condition.

## Review

Pathophysiology of postpartum left main spontaneous coronary artery dissection

Explanation of SCAD

SCAD is a relatively uncommon but progressively acknowledged underlying cause of acute coronary syndrome, particularly among young individuals without pre-existing cardiovascular conditions. This phenomenon involves a tear within the intricate layers of the coronary artery wall. This disruption leads to a false lumen, a separated channel within the artery, which can exert a range of adverse effects on blood flow dynamics and arterial integrity. Depending on the severity and location of the dissection, this abnormal channel can lead to blood flow obstruction or accumulation of blood within the artery's wall, causing the formation of a hematoma [1,7].

The consequences of SCAD include the potential for compromised blood supply to the heart muscle, leading to myocardial ischemia or even myocardial infarction. This condition poses significant challenges to the heart's functioning and can result in a spectrum of symptoms, including chest pain, shortness of breath, palpitations, and fatigue. SCAD often defies the stereotypical cardiac profile, affecting young and otherwise healthy individuals. This unique demographic aspect complicates diagnosing and managing SCAD [8,9].

SCAD disproportionately affects women, constituting a significant proportion of reported cases. This gender predilection is particularly pronounced in cases involving the left main coronary artery, where women's susceptibility is more pronounced. This emphasizes the need for a gender-specific lens when considering cardiovascular health and underscores the importance of understanding the intricate interplay between hormonal, genetic, and physiological factors in the pathogenesis of SCAD [10,11].

Unique Characteristics and Triggers of Postpartum SCAD

Postpartum SCAD stands out as a distinctive subset of SCAD cases due to its occurrence within the weeks following childbirth. This temporal association suggests hormonal and physiological changes during pregnancy and postpartum contribute to its pathogenesis. The abrupt hormonal shifts, especially the rapid decline in estrogen and progesterone levels after delivery, have been proposed as potential triggers for arterial wall fragility. The mechanical stress exerted on coronary arteries during labor and delivery may also contribute to developing postpartum SCAD [12,13]. Moreover, psychological stress, sleep deprivation, and alterations in blood volume during the postpartum period could further exacerbate the vulnerability of coronary arteries, increasing the risk of dissection. The interaction between these triggers and individual susceptibility factors may underlie the increased incidence of SCAD during this specific period [14].

Hormonal and Physiological Changes During Pregnancy and Their Potential Influence

Pregnancy induces significant physiological changes to accommodate the growing fetus and maintain maternal homeostasis. Estrogen and progesterone are pivotal in these adaptations, affecting the cardiovascular system. Estrogen, for instance, exerts vasodilatory effects and influences endothelial function. Progesterone affects smooth muscle tone and may impact arterial wall integrity [15].

During pregnancy, the myocardium experiences increased workload and cardiac output, leading to cardiac remodeling and changes in the coronary circulation. These changes might alter the biomechanical properties of coronary arteries and render them more susceptible to injury, particularly in the presence of hormonal fluctuations [16]. However, the exact mechanisms by which these hormonal and physiological changes contribute to the development of postpartum SCAD remain an active area of investigation. Unraveling these intricate interactions could provide insights into preventive strategies and targeted interventions for at-risk women.

Clinical presentation and diagnosis

Common Symptoms and Signs of PLMSCAD

The clinical presentation of PLMSCAD is marked by a diverse range of symptoms, a characteristic that can complicate its timely and accurate diagnosis. This is primarily because these symptoms often mimic other cardiac conditions, creating a diagnostic challenge for healthcare professionals. Individuals with PLMSCAD may exhibit signs that include chest pain or discomfort, shortness of breath, palpitations, fatigue, and sometimes even syncope [14].

These symptoms, although indicative of potential cardiac distress, can also be non-specific and common in various other conditions. This overlap makes it difficult for clinicians to identify PLMSCAD promptly. The inherent variability in how these symptoms manifest further complicates the diagnostic process. Additionally, the presentation can be particularly misleading during the postpartum period due to the prevalence of fatigue and sleep deprivation. Women in the postpartum phase commonly face challenges such as sleep disturbances and hormonal fluctuations, which can contribute to discomforts that overlap with PLMSCAD symptoms [17].

Recognizing the complex interplay between childbirth-related physiological changes, hormonal shifts, and potential underlying cardiac conditions necessitates a cautious approach in diagnosing PLMSCAD. The similarity of symptoms to other postpartum physiological changes, such as fluid retention and hormonal fluctuations, poses a significant hurdle in distinguishing between benign and potentially life-threatening conditions. Consequently, healthcare providers must adopt a comprehensive evaluation strategy that integrates clinical judgment, thorough medical history analysis, and advanced diagnostic techniques to identify PLMSCAD and initiate appropriate interventions promptly [18].

Challenges in Diagnosing PLMSCAD: Differentiation From Other Cardiac Conditions

Diagnosing PLMSCAD is intricate due to its relatively low incidence and similar symptoms to other cardiovascular disorders. It's critical to differentiate PLMSCAD from conditions like myocardial infarction, coronary artery spasms, and stress-induced cardiomyopathy (Takotsubo syndrome). The lack of specific biomarkers for PLMSCAD further complicates diagnosis [19].

Moreover, the unique demographic of postpartum women adds another layer of complexity. Healthcare providers must be vigilant to avoid misdiagnosis and ensure timely intervention. A high index of suspicion is necessary when evaluating young, otherwise, healthy women presenting with acute coronary symptoms in the postpartum period [20].

Role of Various Diagnostic Methods

Diagnostic imaging plays a pivotal role in confirming the diagnosis of PLMSCAD and guiding appropriate management strategies. Coronary angiography remains the gold standard for assessing coronary artery anatomy and identifying dissections. It can reveal the characteristic "double-lumen" appearance, often indicative of SCAD [21].

Intravascular imaging techniques, such as intravascular ultrasound (IVUS) and optical coherence tomography (OCT), offer high-resolution views of the coronary artery walls, accurately assessing dissection severity and location. These methods are precious in cases where more than angiography alone might be needed to provide sufficient detail [22].

Non-invasive imaging, such as cardiac magnetic resonance imaging (MRI) and computed tomography angiography (CTA), also contribute to the diagnostic process by providing additional anatomical information and aiding in assessing myocardial viability [23]. In cases where PLMSCAD is suspected but not confirmed through initial imaging, serial imaging and close clinical monitoring may be necessary, considering the potential for spontaneous dissection healing over time.

Current management strategies

Initial Stabilization and Management in Acute Cases

In the context of acute cases of PLMSCAD, the urgency for prompt and effective stabilization cannot be overstated. Achieving and maintaining hemodynamic stability is a primary concern in these critical scenarios. This involves swiftly implementing a series of carefully orchestrated medical interventions and close monitoring to mitigate the potentially life-threatening consequences of the condition [24].

Ensuring adequate oxygen supply is a foundational step in the management process. Oxygen supplementation alleviates the strain on the compromised cardiovascular system, promoting oxygenation of vital organs and tissues. Addressing pain relief is paramount for patient comfort and managing the physiological stress exacerbating the condition. Administering appropriate pain management strategies contributes to the overall stability of the patient's cardiovascular state [25].

Equally integral is the meticulous management of fluid levels. Careful fluid management is crucial to avoid overloading the heart and further burdening the compromised circulation. Monitoring and regulating fluid intake help maintain optimal intravascular volume and pressure, facilitating efficient blood circulation and cardiac function [26].

Given the inherent potential for rapid deterioration in cases of PLMSCAD, early recognition is an indispensable aspect of effective management. Timely identification of symptoms and accurate diagnosis sets the stage for immediate intervention. Recognizing the criticality of the situation, the seamless transfer of the patient to a specialized cardiac care facility becomes a pivotal step. These facilities are equipped with the expertise, resources, and infrastructure required to address the intricate challenges posed by PLMSCAD [27].

Medical Management

Medical management is critical in stabilizing patients with PLMSCAD and preventing complications. Antiplatelet agents, such as aspirin and P2Y12 inhibitors, are commonly administered to reduce the risk of thrombosis and subsequent ischemic events. Beta-blockers control heart rate and blood pressure, minimizing the coronary artery's mechanical stress. Angiotensin-converting enzyme (ACE) inhibitors may be employed to manage blood pressure and reduce myocardial workload [28]. The selection of medications should be tailored to the patient's characteristics, comorbidities, and response to treatment. Close monitoring and titration are essential to achieve optimal therapeutic outcomes while minimizing potential adverse effects.

Interventional Approaches: Percutaneous Coronary Intervention (PCI) vs. Coronary Artery Bypass Grafting (CABG)

The choice between PCI and CABG depends on the extent of dissection, the involvement of side branches, and the patient's overall condition. In some cases, PCI with stent placement may be feasible to restore blood flow and alleviate ischemia. However, the fragility of the arterial wall in PLMSCAD poses challenges in stent placement, and the risk of extension of dissection must be carefully considered [29].

CABG might be preferred when there is extensive involvement of the left main coronary artery, multiple vessel dissections, or PCI is deemed too risky. CABG allows for creating a stable bypass conduit, avoiding the complications associated with stent placement in fragile arteries [29]. Commonly used arteries for CABG in cases of postpartum left main spontaneous coronary artery dissection include the internal thoracic artery (ITA) and radial artery. These arteries are often chosen due to their durability and suitability for grafting [30].

Role of Multidisciplinary Teams

The successful management of PLMSCAD necessitates a multidisciplinary approach combining expertise from various medical specialties. Given the complexity of PLMSCAD, various healthcare professionals must work cohesively to craft and execute a comprehensive treatment plan, including cardiologists, interventional cardiologists, cardiac surgeons, obstetricians, anesthesiologists, and other specialists [30].

This collaborative approach ensures that the diverse aspects of each patient's case are carefully considered. Each specialist brings a unique perspective, contributing insights that collectively shape a well-informed treatment strategy. Cardiologists and interventional cardiologists bring their expertise in cardiovascular health and intervention techniques, while cardiac surgeons contribute their insights into surgical options when necessary. Obstetricians provide valuable input on the timing of interventions, considering the potential impact on ongoing pregnancies or future fertility desires [31].

Close coordination between the cardiovascular and obstetric teams is vital in addressing the intricacies of PLMSCAD management. Decisions regarding interventions must carefully balance maternal health considerations with the patient's and the postpartum's well-being. The timing of interventions, the choice of treatments, and the potential effects on subsequent pregnancies require thorough discussion and joint decision-making. The multidisciplinary approach ensures that no facet of the patient's condition is overlooked, leading to comprehensive, well-rounded treatment plans that optimize outcomes [32]. Moreover, this collaborative framework extends beyond medical considerations. The psychological, emotional, and ethical dimensions of managing PLMSCAD are addressed more effectively when multiple perspectives are integrated. The pooling of expertise also promotes a culture of shared learning and continuous improvement, fostering innovative solutions and refining management strategies over time.

Outcomes and prognosis

Short-Term and Long-Term Outcomes After PLMSCAD

The outcomes following PLMSCAD can vary widely, with implications for the affected women's short-term recovery and long-term prognosis. In the short term, successful intervention and stabilization can significantly improve symptoms and myocardial perfusion. However, the risk of complications, such as myocardial infarction or arrhythmias, remains a concern [33].

Various factors influence long-term outcomes, including the extent of the dissection, side branches' involvement, treatment strategy choice, and the patient's overall health. Some women experience complete arterial wall healing over time, while others may be left with residual stenosis or ongoing ischemic symptoms. The long-term risk of recurrence is also a consideration, emphasizing the importance of ongoing follow-up care [34].

Factors Influencing Prognosis

The prognosis of PLMSCAD is intricately linked to the characteristics of the dissection itself, including its potential spread to specific coronary arteries or branches. The extent of dissection plays a pivotal role, with those involving major coronary branches posing a higher risk of adverse outcomes. Dissections that extend into significant arteries, such as the left anterior descending artery, can precipitate more severe ischemic events, exacerbating the overall prognosis [33]. Furthermore, the severity of arterial compromise is a critical factor. Patient outcomes are influenced by the clinical presentation during diagnosis, existing comorbidities, and response to treatment. Timely and appropriate interventions significantly contribute to better outcomes, underscoring the importance of swift and effective management.

Impact on Future Pregnancies and Maternal Health Considerations

For women who survive a PLMSCAD episode, the impact on future pregnancies and maternal health is a significant consideration. The cardiac implications of PLMSCAD, coupled with the stresses of pregnancy, may pose unique challenges. Women who have experienced PLMSCAD are often advised to consult with a multidisciplinary team of cardiologists and obstetricians before planning future pregnancies. The timing of subsequent pregnancies, the need for specialized monitoring, and potential interventions should be thoroughly discussed to ensure the best possible outcomes for both the mother and the fetus [35]. Furthermore, the long-term effects of PLMSCAD on maternal health, including the risk of cardiovascular events and the development of coronary artery disease, require continuous vigilance and appropriate risk management strategies.

Challenges in management

Limited Data and Studies Specific to PLMSCAD

One of the significant challenges in managing PLMSCAD is the scarcity of dedicated research and clinical studies focused explicitly on this condition. The rarity of PLMSCAD cases makes it difficult to gather substantial data to inform evidence-based management strategies. Most of the insights into management are extrapolated from broader studies on SCAD and acute coronary syndromes. More specific data is needed to establish standardized protocols and guidelines tailored to PLMSCAD [30].

Variability in Presentation and Response to Treatment

The variability in presentation and response to treatment poses another hurdle in managing PLMSCAD. The clinical spectrum of symptoms, the severity of arterial involvement, and the individual patient's characteristics contribute to a diverse landscape. What works effectively in one case might not be suitable for another. This heterogeneity underscores the importance of personalized medicine in managing PLMSCAD, where treatment plans must be tailored to the patient's unique profile [36].

Ethical Considerations in Managing Postpartum Women With Cardiac Conditions

Managing postpartum women with cardiac conditions, including PLMSCAD, raises ethical considerations that demand careful attention. Balancing the potential risks and benefits of interventions during a critical phase of maternal care is complex. Decisions surrounding medical interventions, particularly regarding future pregnancies, must be made collaboratively with the patient, considering her values, preferences, and long-term health goals. Ethical dilemmas also emerge when balancing the mother's and fetus’s well-being during subsequent pregnancies [37]. Additionally, the psychological and emotional impact of PLMSCAD on postpartum women cannot be understated. Providing appropriate counseling, support, and mental health resources is integral to holistic care.

Genetic predispositions and risk factors in PLMSCAD

The etiology of PLMSCAD is complex and multifactorial, with genetic predispositions emerging as significant contributors to its occurrence. Several genetic conditions and syndromes have been associated with an increased risk of developing PLMSCAD, shedding light on the underlying mechanisms and potential targets for risk assessment and prevention [10].

Fibromuscular Dysplasia (FMD)

FMD is a non-inflammatory, non-atherosclerotic vascular disorder characterized by abnormal cellular growth in the walls of medium and large arteries. It has been linked to PLMSCAD, suggesting that the structural abnormalities in arterial walls seen in FMD may increase susceptibility to dissection. Identifying FMD as a risk factor highlights the importance of considering underlying vascular conditions in patients presenting with PLMSCAD [11].

Marfan Syndrome

Marfan syndrome is a hereditary connective tissue disorder caused by mutations in the FBN1 gene, leading to abnormal connective tissue synthesis. The impact of Marfan syndrome on the cardiovascular system, particularly aortic complications, is well known. Recent research has indicated a potential association between Marfan syndrome and an increased risk of coronary artery dissections, including those involving the left main coronary artery. The genetic alterations in connective tissue elements could contribute to arterial fragility, predisposing individuals to PLMSCAD [11,12].

Connective Tissue Disorders and Vasculitidies

Other genetic conditions that affect connective tissue integrity, such as Ehlers-Danlos syndrome and various vasculitidies, have also been implicated in PLMSCAD. These conditions can compromise the structural integrity of arterial walls, increasing the susceptibility to spontaneous dissection. The genetic variations associated with these disorders may influence the biomechanical properties of the arterial wall, making it more prone to tear or separation [13].

Sarcoidosis

Sarcoidosis is a granulomatous inflammatory disorder that can affect multiple organs, including the heart. Inflammation in the coronary arteries might weaken the arterial wall, making it more susceptible to dissection. Genetic factors that influence the immune response and inflammation pathways could play a role in predisposing individuals with sarcoidosis to PLMSCAD [14].

Treatments and surgeries for PLMSCAD

The management of PLMSCAD involves a range of treatment approaches, each tailored to the specific characteristics of the dissection, the patient's clinical presentation, and underlying health conditions. The effectiveness of these treatments can vary based on individual factors, the extent of arterial involvement, and the chosen intervention strategy.

Medical Management

Medical management is often the initial approach for stabilizing patients with PLMSCAD. This includes administering medications to manage symptoms, reduce the risk of thrombosis, and alleviate stress on the heart. Commonly used medications include antiplatelet agents (aspirin, P2Y12 inhibitors), beta-blockers to control heart rate and blood pressure, and ACE inhibitors to manage blood pressure and reduce myocardial workload. The effectiveness of medical management depends on factors such as the patient's response to treatment, the severity of symptoms, and the presence of any associated conditions [18-20].

Percutaneous Coronary Intervention

PCI involves the insertion of a catheter into the affected coronary artery to open the blockage and restore blood flow. Stent placement may be considered to stabilize the arterial wall and prevent further dissection or stenosis. However, the success of PCI in PLMSCAD can vary due to the fragility of the arterial wall and the potential for complications such as extension of the dissection or stent-related issues. The decision to perform PCI depends on the patient's clinical condition, the dissection's anatomy, and the interventional team's expertise [21-22].

Coronary Artery Bypass Grafting

CABG involves bypassing the blocked or diseased coronary artery using a healthy blood vessel graft from another body part. CABG is considered when the dissection involves extensive portions of the left main coronary artery, multiple vessel dissections, or when PCI is not feasible. CABG provides a stable conduit for blood flow, avoiding the challenges associated with stent placement in fragile arteries. The success of CABG depends on the complexity of the dissection, the surgical technique employed, and the patient's overall health [23,24].

Conservative Management

In some cases, a conservative approach involving close monitoring and medical management may be chosen, especially if the dissection is not causing severe ischemia or other complications. Serial imaging and clinical follow-up are essential to assess the progression of the dissection and the patient's response to treatment. Conservative management may be preferred for patients with less severe dissections or those who are not suitable candidates for more invasive interventions [24].

Effectiveness and Follow-Up

The effectiveness of treatments and surgeries for PLMSCAD can vary widely due to the uniqueness of each case. Success is measured by factors such as symptom relief, blood flow restoration, complications prevention, and long-term outcomes. Regular follow-up appointments, imaging studies, and ongoing assessments are crucial to monitor the progression of the dissection, identify any recurrent symptoms, and adjust the treatment plan as needed [24].

Emerging research and future directions

Recent Advancements in Understanding PLMSCAD

Recent years have witnessed notable progress in unraveling the complexities of PLMSCAD. Advancements in imaging technologies, such as IVUS and OCT, have allowed for more precise visualization of arterial wall characteristics, aiding in accurate diagnosis and treatment planning. These imaging techniques have offered insights into the morphological features of dissections and their evolution over time [38]. Moreover, advances in genetics and molecular biology have shed light on potential predisposing factors for PLMSCAD. Improved understanding of the pathophysiology, including the role of hormonal changes and mechanical stresses during pregnancy, has contributed to a more comprehensive picture of the condition.

Potential Targets for Future Research

Future research on PLMSCAD promises to address critical knowledge gaps and refine management strategies. Genetic factors are exciting, as specific gene mutations might contribute to arterial fragility and predisposition to dissection. Exploring hormonal influences, such as the interplay between estrogen, progesterone, and arterial integrity, could yield insights into preventive measures or targeted interventions.

Long-term follow-up studies are essential for understanding the trajectory of PLMSCAD outcomes beyond the acute phase. Tracking the risk of recurrence, the impact on future pregnancies, and the potential development of coronary artery disease will inform ongoing management strategies.

Importance of Collaborative Research Efforts and Data Sharing

Collaborative efforts are central to advancing our understanding of PLMSCAD. Given the condition’s rarity, pooling data and sharing experiences across multiple institutions are vital for accumulating sufficient knowledge to guide clinical practice. Collaborative research initiatives facilitate larger sample sizes, enhancing the statistical power of studies and enabling more robust conclusions.

Furthermore, the multidisciplinary nature of PLMSCAD management calls for effective communication and collaboration among cardiologists, obstetricians, geneticists, psychologists, and other healthcare professionals. These collaborations foster a holistic approach that considers the medical aspects and the psychological and emotional well-being of affected women.

Patient education and support

The Role of Patient Education in Early Recognition of Symptoms

Patient education plays a crucial role in the early recognition and timely management of PLMSCAD. Empowering women with knowledge about the condition's symptoms and risk factors enables them to seek medical attention promptly when experiencing cardiac-related symptoms. Given the atypical nature of symptoms and the potential overlap with postpartum changes, awareness is paramount in avoiding delays in diagnosis and treatment.

Healthcare providers should emphasize the importance of seeking immediate medical attention for any concerning symptoms, even if they seem unrelated to heart issues. Providing educational materials, workshops, and online resources can help disseminate accurate information and enhance awareness among women, their families, and healthcare professionals.

Support Networks and Resources for Women With PLMSCAD

A diagnosis of PLMSCAD can be overwhelming, and women may experience physical, emotional, and psychological challenges. Establishing support networks and providing resources for women affected by PLMSCAD is essential for promoting overall well-being and facilitating the adjustment to life after diagnosis and treatment.

Patient support groups, both in-person and online, offer a platform for women to connect with others who share similar experiences. These networks provide a space for sharing stories, exchanging coping strategies, and seeking advice from those who have navigated the complexities of PLMSCAD. Online platforms also enable access to information, research updates, and expert insights.

Medical institutions and healthcare providers can contribute to support efforts by collaborating with patient advocacy organizations, organizing patient-centered events, and ensuring that relevant resources are readily accessible. Mental health support is equally critical, as the emotional toll of PLMSCAD can impact a woman's overall quality of life. Providing access to counseling and psychological services can help women manage the psychological challenges associated with the condition.

Future Directions: Risk Assessment and Personalized Care

Identifying these genetic predispositions and risk factors is instrumental in shaping risk assessment strategies and personalized care for individuals at risk of developing PLMSCAD. Genetic testing, when feasible, could help identify individuals with underlying genetic conditions that increase their vulnerability to arterial dissections. This knowledge could inform treatment decisions, surveillance plans, and family counseling regarding the inheritance patterns of these genetic conditions.

Additionally, understanding the genetic basis of PLMSCAD could lead to developing targeted therapies or preventive strategies. Researchers may identify potential therapeutic targets to strengthen the arterial wall or mitigate the dissection risk by uncovering the specific molecular pathways involved in arterial fragility.

## Conclusions

In conclusion, the intricate nature of PLMSCAD underscores the complex challenges that healthcare providers and patients must navigate. This review article has illuminated the unique pathophysiology, diagnostic intricacies, evolving management strategies, and potential research directions. Reflecting on the insights shared, it becomes evident that PLMSCAD requires a multidisciplinary approach, collaboration, and ongoing research efforts. By recognising the importance of early recognition, tailored interventions, and comprehensive patient support, healthcare professionals can contribute to improved outcomes and enhanced maternal well-being. The journey to further understand, effectively manage, and support women affected by PLMSCAD continues to advance maternal healthcare, reducing morbidity and mortality and ensuring mothers' and their families' health and vitality.
